# Factors affecting infant mortality in the general population: evidence from the 2016 Ethiopian demographic and health survey (EDHS); a multilevel analysis

**DOI:** 10.1186/s12884-020-03002-x

**Published:** 2020-05-15

**Authors:** Adhanom Gebreegziabher Baraki, Temesgen Yihunie Akalu, Haileab Fekadu Wolde, Ayenew Molla Lakew, Kedir Abdela Gonete

**Affiliations:** 1 Department of Epidemiology and Biostatistics, University of Gondar, College of Medicine and Health Sciences, Institute of Public Health, Gondar, Ethiopia; 2 Department of Human Nutrition, University of Gondar, College of Medicine and Health Sciences, Institute of Public Health, Gondar, Ethiopia

**Keywords:** Infant mortality, Risk factors, Ethiopia

## Abstract

**Background:**

Infant mortality is one of the leading public health problems globally; the problem is even more staggering in low-income countries. In Ethiopia seven in ten child deaths occurred during infancy in 2016. Even though the problem is devastating, updated information about the major determinants of infant mortality which is done on a countrywide representative sample is lacking. Therefore, this study was aimed to identify factors affecting infant mortality among the general population of Ethiopia, 2016.

**Methods:**

A Community-based cross-sectional study was conducted in all regions of Ethiopia from January 18 to June 27, 2016. A total of 10,641 live births were included in the analysis. Data were analyzed and reported with both descriptive and analytic statistics. Bivariable and multivariable multilevel logistic regression models were fitted by accounting correlation of individuals within a cluster. Adjusted odds ratio (AOR) with 95% confidence interval was reported to show the strength of the association and its significance.

**Results:**

A total of 10,641 live-births from the Ethiopian demographic and health survey (EDHS) data were included in the analysis. Being male infant (AOR = 1.51; 1.25, 1.82), Multiple birth (AOR = 5.49; 95% CI, 3.88–7.78), Preterm (AOR = 8.47; 95% CI 5.71, 12.57), rural residents (AOR = 1.76; 95% CI; 1.16, 2.67), from Somali region (AOR = 2.07; 1.29, 3.33), Harari (AOR = 2.14; 1.22, 3.75) and Diredawa (AOR = 1.91; 1.04, 3.51) were found to be statistically significantly associated with infant mortality.

**Conclusion:**

The study has assessed the determinants of infant mortality based on EDHS data. Sex of the child, multiple births, prematurity, and residence were notably associated with infant mortality. The risk of infant mortality has also shown differences across different regions. Since infant mortality is still major public health problem interventions shall be done giving more attention to infants who were delivered multiple and who are preterm.

## Background

Over the past two decades, the world made substantial progress in reducing infant mortality. In 2017, an estimated 6.3 million children and young adolescents died due to mostly preventable causes. Children under the age of 5 years accounted for 5.4 million of these deaths, with 2.5 million deaths occurring in the first month of life, 1.6 million between age 1–11 months, and 1.3 million between age 1–4 years [1]. According to studies done from 56 countries, infant mortality was one of the global public health concern and accounts for 144 deaths per 1000 live births [2].

In Sub-Saharan Africa, infant mortality has declined from 182 to 58 deaths per 1000 live births from 1990 to 2017. However, Globally, in 2017, half of all deaths under 5 years of age took place in sub-Saharan Africa, and another 30% in Southern Asia [1]. According to the 2016 Ethiopian Demographic and Health Survey (EDHS), the infant mortality rate was 48 deaths per 1000 live births, this constitutes seven in ten under-five deaths in the country [3]. Studying infant mortality is helpful to improve child health and maternal health as well [4].

Across the globe, different studies have been conducted to identify factors affecting infant mortality. Among these not attending antenatal care follow-up [5, 6], wealth index, birth interval [7], maternal age, maternal education [8, 9], domestic violence [10], health-related behavior like delaying in seeking health care [11], high poverty and living rural areas [12], and having metabolic disorders [13] were identified.

Even though the problem is high updated information about the major determinants of infant mortality which is done on a countrywide representative sample is lacking in Ethiopia. Therefore, this study was aimed to investigate determinants of infant mortality in Ethiopia, to identify problems to be focused on in order to sustain the reduction of infant mortality and improve survival.

## Methods

### Study design and setting

The data was obtained after permission is found from the Measure demographic and health survery (DHS) international program (https://dhsprogram.com/). EDHS 2016 is a community-based cross-sectional study that is conducted from January 18, 2016, to June 27, 2016. Ethiopia is situated in the Horn of Africa covering an area around 1.1 million square kilometers. Ethiopia is structured into nine regional states namely Benishangul-Gumuz, Tigray, Oromia, Afar, Amhara, Somali, Southern Nations Nationalities and Peoples (SNNP), Gambela, Harari and two city administrations, Addis Ababa and Dire Dawa. From the total population of the country, only 19% live in urban areas and more than 82% of the country’s total population lives in the regional states of Amhara, SNNP, and Oromia.

### EDHS data and collection procedure

The study used secondary data released by the central statistical agency (CSA). This is the fourth national representative survey done at the country level after the first three surveys conducted in 2000, 2005 and 2011. The primary aim of 2016 EDHS was to provide up-to-date information about the key demographic and health indicators. Both men and women aged 15–59 years were interviewed. Data was also collected from mothers or care-takers of live-born infants in the 5 years preceding the date of the interview. For this study, we have extracted data from the EDHS 2016 which was collected from eligible women 15–49 years of age and who were permanent residents of the selected households. The data was collected using a structured pretested questioner. Training was given for the data collectors before the start of the data collection.

### Sample size, and sampling technique

The study included a total of 10,641 live births born from mothers who were interviewed about births in the preceding 5 years before the survey. We included only women in the reproductive age, from 15 to 49 years old.

The 2016 EDHS sample was stratified and selected in two stages. Each region was stratified into urban and rural areas, which yielded 21 sampling strata. Samples of enumeration areas (EAs) were selected independently in each stratum in two stages. Implicit stratification and proportional allocation were done at each of the lower administrative levels by sorting the sampling frame within each sampling stratum before sample selection. In the first stage, 645 EAs (202 from urban and 443 from rural) were selected with a proportional allocation of EA with an independent selection in each sampling stratum. A sampling frame was prepared for each selected EAs by listing households, this was used to select households in the second stage. In the second stage of selection, a fixed number of 28 households per cluster were selected with an equal probability systematic selection from the sampling frame. The survey interviewer interviewed only pre-selected households [3].

### Variables of the study

The outcome variable for this study was infant mortality which was defined as the death of a live birth before the first birthday and it was estimated based on the information collected from the birth history section of the questioner which was administered to individual women.

The individual-level factors included in this study were; current age of the mother, age at first birth, sex of the household head, marital status, household wealth index, educational status both for the mother and the father, occupational status of the mother, size of the child at birth, number of living children in the household, child sex, birth order, duration of pregnancy, preceding birth interval and whether the child is of multiple births or not, place of delivery, number of antenatal care (ANC) visits, number of tetanus toxoid (TT) injections during pregnancy and mode of delivery. Wealth index for the households was originally classified into five categories by DHS which was done using principal component analysis but for easy interpretation, we reclassified the wealth scores into three categories (poor, medium, rich)***.*** The community-level factors included in this study were residence and region.

### Data processing and analysis

After data cleaning was done descriptive analysis was done using frequencies and percentages. A Chi-square test was done for all categorical independent variables to check the assumptions. Both bivariable and a multivariable multilevel logistic regression model was fitted by taking the clusters as a random effect. Variables having *p*-value < 0.2 from the bivariable analysis were included in the final multivariable model. Variables having a p-value < 0.05 from the multi-variable model were considered as having a significant association with infant mortality. Fixed effects were presented in terms of odds ratio with its 95% CI. The result of the random effect variation was checked using the intracluster correlation (ICC). The model goodness of fitness was checked using deviance information criteria (DIC). STATA version 14 was used for data analysis.

## Results

### Socio-demographic characteristics of study participants

Of the total 10,641 live-births included in the analysis, 8667(81.5%) were the rural residents. The mean age of respondents was 29.23(±6.5 SD). The majority, 3161(29.7%), of the mothers were in the age group of 25–29 years. About two-thirds (6838) and half (5003) mothers and their partners did not have any formal education respectively. The larger proportion, 6307(59.3%), of the mothers were housewives and a large number, 9903 (93.06%), of the participants were married. More than half, 5775(54.3%), of the infants were from households categorized in poor income (Table 1).

**Table 1:** Sociodemographic characteristics of study participants EDHS 2016

Variable	Frequency	Percent
**Age (mean age:29.23 ± 6.5 SD)**		
15–19	404	3.8
20–24	2171	20.4
25–29	3161	29.71
30–34	2360	22.18
35–39	1697	15.95
40–44	647	6.08
45–49	201	1.89
**Residence**		
Urban	1974	18.55
Rural	8667	81.45
**Mothers educational status**		
No education	6838	64.26
Primary	2678	25.17
Secondary	734	6.9
Higher	391	3.67
**Husbands educational status**		
No education	5003	49.99
Primary	3220	32.17
Secondary	1015	10.14
Higher	770	7.69
**Mothers occupation**		
Housewife	6307	59.27
Employed	4334	40.73
**Marital status**		
Never in union	61	0.57
Married	9903	93.06
Living with a partner	105	0.99
Widowed	135	1.27
Divorced	328	3.08
Separated	109	1.02
**Wealth index**		
Poor	5775	54.27
Medium	1466	13.78
Rich	3400	31.95
**Religion**		
Orthodox	3082	28.96
Catholic	72	0.68
Protestant	1862	17.50
Muslim	5442	51.14
Traditional	103	0.97
Other	80	0.75
**Region**		
Tigray	1033	9.71
Afar	1062	9.98
Amhara	977	9.18
Oromia	1581	14.86
Somali	1505	14.14
Benshangul	879	8.26
SNNPR	1277	12.0
Gambela	714	6.71
Harari	605	5.69
Addis Ababa	461	4.33
Dire Dawa	574	5.14
**Sex of Household head**		
Male	8383	78.78
Female	2258	21.22

### Obstetric related characteristics

More than half, 5940(55.8%) of mothers give their first birth when their age was between 15 and 19. Almost two-thirds (63%) of mothers give their second to sixth births and about 3230 mothers have 3–4 live births. The average number of live births was 3 (IQR: 2, 5). The majority of live-births had an average size when they were born. More than half, 7271(68.33%) of the mothers delivered at home, and most, 10,391(97.65%), of them gave birth at term (Table 2).

**Table 2:** Obstetric characteristics of participants EDHS 2016

Variable	Frequency	Percent
**Age of respondent at 1st birth: mean = 19.1 (±3.67 SD)**		
11–14	622	5.85
15–19	5940	55.82
20–24	3187	29.95
25–29	719	6.76.
30–34	151	1.42
35+	22	0.21
**Size of the child at birth**		
Large	3214	30.20
Average	4419	41.53
Small	2890	27.16
Don’t know	118	1.11
**Birth order**		
1	2167	20.36
2–3	3338	31.37
4–6	3385	31.81
7+	1751	16.46
**Number of living children**		
0	76	0.71
1–2	3652	34.32
3–4	3230	30.35
5+	3683	34.61
**Child sex**		
Male	5483	51.53
Female	5158	48.47
**Twins birth**		
Yes	278	2.61
No	10,363	97.39
**Duration of pregnancy**		
Term	10,455	98.26
Preterm	186	1.74
**Place of delivery**		
Home	7271	68.33
Gov’t hospital & HC	2832	26.61
Health post	191	1.79
Private hospital, clinic &sector	263	2.47
NGO health facility	84	0.79
**Number of TT injections during pregnancy (7193)**		
0–1	4203	58.43
≥ 2	2990	41.57
**Delivery by CS**		
Yes	305	2.87
No	10,336	97.13
**Number of ANC visits during pregnancy (*n*** **= 7193)**		
No visit	2481	34.49
1–3 visit	2092	29.08
≥ 4 visit	2601	36.16
Don’t know	19	0.26

### Determinants of infant mortality

Based on the multivariable multilevel logistic regression output residence, region, child sex, multiple births, and duration of pregnancy were found to be significantly associated with infant death.

The odds of infant death among male children was 1.51 times higher as compared to females (AOR = 1.51; 1.25, 1.82). The odds of death was 5.49 (AOR = 5.49; 95% CI, 3.88–7.78) times higher among multiple births as compared to singletons. Preterm infants were 8.47(AOR = 8.47; 95% CI 5.71, 12.57) times more likely to die as compared to infants born at term. The risk of infant mortality among rural residents was 1.76 (AOR = 1.76; 95% CI; 1.16, 2.67) times higher when compared to urban residents. Mortality risk was higher among children born in the east of Ethiopia; the risk of infant death among infants born in Somali (AOR = 2.07; 1.29, 3.33), Harari (AOR = 2.14; 1.22, 3.75) and Diredawa (AOR = 1.91; 1.04, 3.51) was higher as compared to Tigray region (Table 3).

**Table 3:** Multilevel multivariable logistic regression output for determinants of infant mortality among infants in Ethiopia, 2016

Variable	Crude OR (95% CI)	Model I AOR (95% CI)	Model II AOR (95% CI)	Model III AOR (95% CI)
**Age**				
15–19	1.00		1.00	1.00
20–24	0.94 (0.59, 1.49)		0.89 (0.55,1.44)	0.92 (0.57, 1.48)
25–29	0.74 (0.47, 1.16)		0.71 (0.43,1.18)	0.77 (0.46, 1.29)
30–34	0.64 (0.40, 1.02)		0.57 (0.33,1.00)	0.66 (0.37, 1.16)
35–39	0.81 (0.50, 1.31)		0.64 (0.35,1.14)	0.75 (0.42, 1.36)
40–44	0.74 (0.42, 1.30)		0.56 (0.28,1.10)	0.66 (0.33, 1.32)
45–49	1.28 (0.65, 2.54)		1.02 (0.46,2.27)	1.22 (0.55, 2.72)
**Mothers’ educational Status**				
No education	1.49 (1.05, 2.11)		1.51 (0.98,2.30)	1.25 (0.80, 1.93)
Primary	1.29 (0.88, 1.89)		1.26 (0.83,1.90)	1.12 (0.73, 1.71)
Secondary and above	1.00		1.00	1.00
**Wealth Index**				
Poor	1.44 (1.15, 1.79)		1.20 (0.93,1.55)	1.01 (0.76, 1.35)
Medium	1.11 (0.81, 1.53)		0.93 (0.67,1.31)	0.83 (0.58, 1.18)
Rich	1.00		1.00	1.00
**Sex of Household head**				
Male	1.26 (0.99, 1.60)		1.27 (0.99,1.62)	1.28 (0.99, 1.65)
Female	1.00		1.00	1.00
**Birth order**				
1	0.94 (0.70, 1.25)		1.00 (0.64,1.56)	1.13 (0.73, 1.77)
2–3	0.77 (0.59, 1.01)		0.80 (0.55,1.17)	0.89 (0.61, 1.30)
4–6	0.79 (0.60, 1.02)		0.84 (0.62,1.15)	0.89 (0.65, 1.22)
7+	1.00		1.00	1.00
**Child sex**				
Male	1.52 (1.27, 1.83)		1.50 (1.24, 1.82)	1.51 (1.25, 1.82)*
Female	1.00		1.00	1.00
**Twin birth**				
No	1.00		1.00	1.00
Yes	5.44 (3.91, 7.58)		5.53 (3.90,7.83)	5.49 (3.88, 7.78)*
**Duration of pregnancy**				
Pre-term	8.16 (5.61, 11.87)		7.80 (5.29,11.50)	8.47 (5.71, 12.57)*
Term	1.00		1.00	1.00
**Place of delivery**				
Home	1.00		1.00	1.00
Government hospital/health center	0.77 (0.62, 0.97)		0.80 (0.62,1.03)	0.89 (0.68, 1.17)
Health post	0.74 (0.34, 1.60)		0.77 (0.35,1.70)	0.78 (0.36, 1.72)
Private and NGO HF	0.67 (0.37, 1.23)		0.68 (0.35,1.33)	0.76 (0.38, 1.52)
**Residence**				
Urban	1.00	1.00		1.00
Rural	1.74 (1.29, 2.33)	1.83 (1.30, 2.57)		1.76 (1.16, 2.67)*
**Region**				
Tigray	1.00	1.00		1.00
Afar	2.02 (1.24, 3.28)	1.96 (1.21,3.18)		1.66 (0.99, 2.76)
Amhara	1.53 (0.92, 2.54)	1.48 (0.89,2.45)		1.34 (0.79, 2.25)
Oromia	1.51 (0.95, 2.42)	1.44 (0.90,2.29)		1.26 (0.78, 2.05)
Somali	2.14 (1.35, 3.37)	2.18 (1.38,3.43)		2.07 (1.29, 3.33)*
Benshangul	1.84 (1.11, 3.06)	1.75 (1.06,2.91)		1.59 (0.94, 2.67)
SNNPR	1.47 (0.90, 2.38)	1.41 (0.87,2.29)		1.42 (0.87, 2.33)
Gambela	1.53 (0.88, 2.64)	1.60 (0.93,2.76)		1.28 (0.73, 2.27)
Harari	1.93 (1.12, 3.33)	2.15 (1.25,3.72)		2.14 (1.22, 3.75)*
Addis Ababa	0.94 (0.47, 1.88)	1.60 (0.75,3.39)		1.3 (0.58, 2.89)
Dire Dawa	1.54 (0.86, 2.77)	1.88 (1.03,3.40)		1.91 (1.04, 3.51)*
**Null Model**				
**Intercept**	0.52 (0.39, 0.71)	0.46 (0.31, 0.66)	0.49 (0.36,0.69)	0.45 (0.30, 0.67)
**Log-Likelihood**	− 2021.15	−2004.79	− 1909.66	− 1898.87
**Deviance**	4042.3	4009.58	3819.32	3797.74

*P-value < 0.05

**Model I:** community-level factor **Model II:** individual-level factor **Model III:** Both community and individual-level factors

## Discussions

The infant mortality rate in Ethiopia was 48 per 1000 live births in 2016. In this study, we have found sex of the child, multiple birth, duration of pregnancy, residence, and region to be significantly associated with infant mortality.

The infant mortality rate (48 per 1000 live birth) in Ethiopia was consistent with the infant mortality rate of World Health Organization (WHO) African regions (51 per 1000 live births), but it was higher than most of the developed countries like United States (5.96 per 1000 live births) [14], Canada (5.2 per 1000 live births) [15] and WHO European region (8 per 1000 live births). However, the infant mortality in Ethiopia was significantly lower than several other countries like Afganistan (110.6 per 1000 live births), Somalia (94.8 per 1000 live births), and Nigeria (69.8 per 1000 live births) [16]. The main reason for this discrepancy could be the difference in socio-economic status, health facility coverage, and other infrastructures.

The odds of infant death were higher among male infants as compared to females. This finding is in line with studies conducted in Kenya [17] and a multicenter study [18] but another study conducted in Brazil found no association between infant death and sex of the baby [8]. The higher risk of death among male infants could be explained by sex differences in genetic and biological makeup, with boys being biologically weaker and more susceptible to diseases and premature death [19].

The risk of death among infants born two or more was higher than singletons, which is supported by other studies conducted in Brazil [8, 20] Kenya [17] and Greece [21, 22]. Having multiple births can be a challenge for the mother to give care which makes the infants at risk of malnutrition and infections. The risk of being low birth weight also increases when there is multiple pregnancy and this affects the children survival [23].

Infants born preterm were more likely to die as compared to the term infants. This result is consistent with other studies [8, 24]. The reason could be respiratory morbidity, feeding difficulties, hyperbilirubinemia, temperature instability, infection, and hypoglycemia which is more common among preterm infants than infants born at term [25–29].

The odds of infant death among rural residents was higher than that of the urban. This finding is consistent with other studies [12, 30]. The reason behind could be the lack of access to health institutions to have ANC follow-ups or lack of media exposure which in turn affects their knowledge and practice of care for the infant [31, 32].

The odds of death among infants living in the eastern part of Ethiopia (Harari, Dire Dawa, and Somali) was higher than infants in Tigray, the northern part of the country. The possible reason could be drug abuse especially chat chewing which is more common in these areas which results in child neglect and poor healthcare-seeking behavior [3]. Mothers who use chat/ abuse drugs are also more likely to deliver a baby with low birth weight due to the effect of chat on the growth of fetus by inhibiting uteroplacental blood flow this, in turn, affects the survival of the baby [33, 34]. These regions are also known for socio-economic vulnerability and food insecurity leading to malnutrition and infant death [35].

The significant determinant of infant mortality in several studies, maternal education [8, 9], was not significantly associated with infant death in this study. The possible reason could be the difference in the content and quality of the education provided across different countries. Besides, the sample size difference could also be another contributor to this discrepancy.

## Conclusions

The study has assessed the determinants of infant mortality based on EDHS data. Sex of the child, multiple births, duration of pregnancy, and residence were significantly associated with infant mortality. The risk of infant mortality has also shown differences across different regions. Since infant mortality is still major public health problem interventions shall be done giving more attention to women with multiple births and infants delivered before term. Expanding the primary health care network in more susceptible regions would also limit infant death.

### Limitations

These study shares limitations of cross-sectional studies, therefore, may not be strong in establishing a cause-effect relationship. We were not directly involved in the data collection which makes us uncertain about the data quality.

## Data Availability

Data is available on https://dhsprogram.com/data/available-datasets.cfm

## Abbreviations

ANC: Antenatal Care
AOR: Adjusted Odds Ratio
CI: Confidence Interval
CSA: Central Statistical Agency
DHS: Demographic and Health Survey
DIS: deviance information criteria
EA: Enumeration Area
EDHS: Ethiopian Demographic and Health Survey
ICC: Intracluster correlation
SD: Standard Deviation
SNNP: Southern Nations Nationalities and Peoples
TT: Tetanus Toxoid
